# Neuro-Behavioral Profile and Toxicity of the Essential Oil of *Dorema ammoniacum* Gum as an Anti-seizure, Anti-nociceptive, and Hypnotic Agent with Memory-enhancing Properties in D-Galactose Induced Aging Mice

**DOI:** 10.22037/ijpr.2020.113738.14458

**Published:** 2020

**Authors:** Reza Jahani, Mona Khoramjouy, Azadeh Nasiri, Maryam Sojoodi Moghaddam, Yousef Asgharzadeh Salteh, Mehrdad Faizi

**Affiliations:** a *Department of Pharmacology and Toxicology, School of Pharmacy, Shahid Beheshti University of Medical Sciences, Tehran, Iran. *; b *Department of Pharmacognosy, School of Pharmacy, Shahid Beheshti University of Medical Sciences, Tehran, Iran.*

**Keywords:** Dorema ammoniacum gum, Epilepsy, Insomnia, Memory, GABA_A_

## Abstract

In this study, we focused on the neuro-behavioral profile, toxicity, and possible mechanisms of action of *Dorema ammoniacum* gum essential oil (DAG-EO). For this purpose, passive avoidance and Y-maze tests were performed to evaluate the potential effect of DAG-EO in the attenuation of memory impairment induced by 49 days administration of D-galactose and acute injection of scopolamine. Anticonvulsant and anti-nociceptive activities of DAG-EO were evaluated in the pentylenetetrazole and ‎maximal electroshock-induced models of seizure and acetic acid-induced writhing tests, respectively. To find the possible mechanism of action, flumazenil and naloxone were used. Furthermore, the possible side effects were determined in the open field, grip strength, and ‎rotarod tests. Our findings supported that 7-day administration of DAG-EO (50 and 100 mg/kg) improves memory impairment induced following administration of D-galactose and scopolamine. It was also revealed that DAG-EO possesses a dose-dependent sedative-hypnotic (100 mg/kg), anticonvulsant (ED_50 _≈ 170 mg/kg), and anti-nociceptive (ED_50 _≈ 175 mg/kg) activities possibly mediated via directly and/or indirectly modulation of GABA_A_ and opioid receptors. No side effect was observed except muscle relaxation which was less than that of diazepam. The output of this study confirms anti-seizure, anti-nociceptive, sedative-hypnotic, and memory-enhancing properties of DAG-EO by modulation of GABA_A_ receptors.

## Introduction

Plants and herbal remedies, even a whole plant or a specific part of the plant, have been widely utilized since ancient times as one of the first sources of therapeutic and healing medicines for the treatment of human diseases and illnesses (1, 2). Over 80,000 plants with medicinal properties are used for medical preparations such as tablets, tinctures, extracts, and ointments. Currently, many natural products derived from plants and medicines with plant-sourced active ingredients or synthetic compounds which mimic a plant-derived compound are prescribed by physicians for prevention, diagnosis, improvement or treatment of physical and mental illnesses (3). *Dorema ammoniacum* D. Don also known as Vosha and the gum-resin, commonly known as gum ammoniacum, have been used in various traditional remedies due to different pharmacological properties such as cytotoxic (4), antibacterial (5), antifungal (6), anticonvulsant (7), acetylcholinesterase inhibitory (8), antioxidant (9), analgesic, and anti-inflammatory activities (10).

Previously, acetylcholinesterase inhibitory activity of some active components of the gum-resin from *Dorema ammoniacum* was reported by Adhami *et al.* (8). There are strong evidences for the involvement of cholinergic and glutamatergic neurons in the etiology of Alzheimer’s disease. Thus, restoring cholinergic function through inhibition of acetylcholinesterase activity will result in the improvement of memory in Alzheimer’s disease (11). It seems that plants with this property have the potential to be used as a therapeutic option in dementia therapy. Motevalian *et al.* investigated the anticonvulsant activity of *Dorema ammoniacum* gum (DAG) aqueous extract in pentylenetetrazole (PTZ) induced model of seizure. In their study, Gamma-aminobutyric acid (GABA) and opioid systems were considered as two possible mechanisms of action since flumazenil and naloxone significantly inhibited changes in latency and duration of seizure produced by DAG (7). The PTZ model of seizure is often associated with petit mal seizures and there is no report on the effect of DAG on the grand mal model of seizure induced by maximal electroshock (MES) (12). The aqueous extract of DAG also revealed analgesic activity in the study conducted by Bakhtiarian* et al.* but the mechanism of action was not investigated during their study (10). 

It seems that the most scientific papers have focused on the pharmacological activity of the DAG extract without considering the mechanism of action, and there is a lack of knowledge about specific pharmacological effects of DAG essential oil (DAG-EO). Therefore, we aimed to evaluate the neuro-pharmacological activities of DAG-EO, revealing the possible mechanisms of action, and investigating the potential side effects using behavioral methods. 

## Experimental

*Materials*

Scopolamine, D-galactose, pentylenetetrazole, pentobarbital, and flumazenil were purchased from Sigma-Aldrich (Steinheim, Germany). Diazepam, celecoxib, and naloxone were kindly donated by Darou Pakhsh Pharmaceutical Factory (Tehran, Iran). Dimethyl sulfoxide (DMSO) was obtained from Merck (Darmstadt, Germany). *Dorema ammoniacum *gum with the voucher specimen no. 8061 was obtained from the Department of Pharmacognosy at Shahid Beheshti University of Medical Sciences (Tehran, Iran). A 500 mL round-bottomed flask connected to a Clevenger apparatus was used for the essential oil extraction by water distillation. The essential oil in the volume of 1.3 mL was obtained from 100 grams of DAG. The density of the essential oil was estimated to be 0.384 g/mL, using the gravimetric method at room temperature. The essential oil was stored in the refrigerator until the day of the experiment. 

*Animals and drug administration*

All experimental procedures were executed in accordance with the institutional procedures of the Animal Experimentation Committee of Shahid Beheshti University of Medical Sciences, Tehran, Iran (Code number: IR.SBMU.PHNM.1395.411). Male C57BL/6J mice (weight, 22-25 g) were used in the D-galactose-induced accelerated aging model and the rest of the experiments were conducted on male NMRI mice (weight, 18-22 g). The mice were kept in the animal house under the same ambient conditions (22 ± 2 ºC, 50% ± 10% humidity) and a 12-h diurnal light cycle with free access to food and water. The animals were randomly divided into groups of ten, and each mouse was used only once. The mice were intraperitoneally treated with the DAG-EO (diluted in sesame oil) in the volume of 5 mL/kg. Scopolamine, D-galactose, pentylenetetrazole, pentobarbital, celecoxib, and naloxone were dissolved in normal saline (0.9%, w/v) and administered in the volume of 10 mL/kg by intraperitoneal (i.p.) route. Diazepam and ‎flumazenil were dissolved in DMSO. The injection volume of diazepam and flumazenil was decreased to a constant volume of 5 mL/kg body weight (i.p.) to reduce the toxicity of DMSO. In all experiments, the animals in the vehicle group received sesame oil.

*Biological activity*

*D-galactose- and scopolamine-induced memory impairment*

The mice were subcutaneously treated with D-galactose at a dose of 100 mg/kg once daily for 49 consecutive days to generate D-galactose-induced aging mice (13). Sham group received normal saline instead of D-galactose. Scopolamine-induced memory impairment was induced by the injection of a single dose of scopolamine (1 mg/kg). Memory-related behavioral responses were measured in the step-through passive avoidance and Y-maze tasks to evaluate the effect of DAG-EO on D-galactose- and scopolamine-induced memory impairment models.

*Step-through passive avoidance test*

DAG-EO (50 and 100 mg/kg) were administered once daily for 7 consecutive days within the last 7 days injection of D-galactose. The step-through passive avoidance test was performed as previously described (14). To summarize, one hour after the last administration of the essential oil, each mouse was placed in the bright compartment of a light-dark apparatus and received a constant current electrical stimulation (50 Hz, 0.2 mA, 2 s) when they entered the dark compartment. On the next day, the mice were again placed into the illuminated part of the apparatus, and latency in the entrance to the dark compartment was recorded. In the scopolamine-induced memory impairment test, the mice were treated with different doses of the essential oil (50 and 100 mg/kg). Following 15 min, they received scopolamine (1 mg/kg) and 15 min later, the step-through passive avoidance test was performed as described above. 

*Y-maze test*

Y-maze apparatus made of three opaque plastic arms (A, B, and C) at a 120° angle from each other and a central zone was used according to the method described by Huh *et al.* (15). One hour after the last administration of DAG-EO in D-galactose-induced memory impaired group and 15 min after the injection of scopolamine in the scopolamine-induced memory impaired mice, each mouse was placed in the central zone of the apparatus and allowed to freely explore the three arms. The number of arm entries was recorded over 10 min, and the percentage of spontaneous alternation was calculated by the following Equation:

Spontaneous alternation% = [(Number of alternations)/(Total arms entries-2)] × 100

*Open field test*

The open field test was performed to evaluate the motor activity of the mice following the administration of DAG-EO (16). A cubic chamber (40 × 40 cm) made of clear Plexiglas wall (40 cm high) was used in this experiment. Locomotion of each mouse was recorded for 10 min in the open field arena, using a digital camera placed above the chamber. The test was performed one hour after the last administration of DAG-EO on day 49 and 24 h later on day 50. An automated tracking system (Ethovision XT software, Noldus, The Netherlands) was used in order to analyze the recorded videos, and total distance movement in cm was considered as the total locomotor activity of the animals.

*Pentobarbital-induced sleep test*

This test was employed to evaluate the hypnotic effect of the essential oil (17). The mice at different groups received DAG-EO (50 and 100 mg/kg), diazepam (2 mg/kg) or vehicle. Pentobarbital at the dose of 60 mg/kg was used for sleep induction 30 min following each treatment, and sleep duration (the duration between loss and recovery of righting reflex) was recorded. 

*Pentylenetetrazole-induced seizures test*

The anticonvulsant activity of the essential oil was evaluated in PTZ and MES induced seizure tests. PTZ test was conducted according to what explained by Mohammadi-Khanaposhtani *et al. *(18). To summarize, the mice were treated with DAG-EO, diazepam as the positive control, and vehicle. The ability of each treatment in the protection of mice against the lethal dose of PTZ (100 mg/kg) was evaluated by recording the number of death following 30 min. Flumazenil (10 mg/kg) and naloxone (1 mg/kg) were used to find out the possible mechanism of action(s).

*Maximal electroshock induced seizures test*

The ability of DAG-EO to prevent MES induced seizures was evaluated in this test. Thirty minutes after the administration of DAG-EO, diazepam or vehicle, an electrical stimulation (10 Hz, 37.2 mA, and 0.3 s) was applied by the apparatus (Borj Sanat, Iran) through the ear electrodes, and the number of mice protected from hind limb tonic extension (HLTE) was recorded following each treatment (19). Flumazenil (10 mg/kg) and naloxone (1 mg/kg) were used to find out the possible mechanism of action(s).

*Acetic acid-induced writhing test*

This is a test to evaluate the analgesic activity of an agent. In this test, any writhing (stretch, tension to one side, an extension of hind legs and finally contraction of the abdomen) was considered as a positive response (20). Writhing was induced by intraperitoneal injection of acetic acid (1% v/v) in the volume of 0.1 mL 30 min after administration of DAG-EO or celecoxib as a positive control at different doses and the writhing episodes were recorded for 20 min. The following equation was used to evaluate the percentage of inhibition against abdominal writhing. In this equation, Nc and Nt refer to the number of constrictions or writhes in the control group and treated group, respectively. 

Protection% = (Nc-Nt)/Nc × 100

*Acute toxicity*

*Non-fatal dose and median lethal dose*

Following acute administration of DAG-EO at different doses, the mice were observed for 3 days. The maximum non-fatal dose (the dose that had not induced any mortality) and median lethal dose (LD_50_) were estimated based on the number of dead mice during the experiment. During 49 days of the experiment, the mice were observed and any change in the weight of animals was recorded. 

*Grip strength test*

Neuromuscular function and the maximal force developed by mice could be assessed in the forelimb grip strength test (21). This test was performed with a grip strength meter (GS 5000, Borj Sanat Co.). Thirty minutes after the administration of DAG-EO (50 and 100 mg/kg), diazepam (2 mg/kg) or vehicle, the mice were placed on the tension pad of the apparatus, and the maximum force (gram) was recorded automatically by the apparatus. Each mouse was tested three times and the averaged value was reported as the final grip strength (22). 

*Rotarod test*

Motor impairing properties of DAG-EO was assessed in the rotarod test. The mice were trained daily for 3 consecutive days on the rotarod apparatus (rod diameter: 3 cm) rotating at a constant speed of 6 rpm. During each training session, the animals were placed on a rotating rod for 3 min with an unlimited number of trials, and only the mice that were able to remain on the rotating rod for 1 min were chosen for the test day. On the test day, the mice were intraperitoneally pretreated with vehicle, DAG-EO, and diazepam (2 mg/kg). After 30 min, the test was conducted and the latency to fall during 120 s was recorded (23). 

*Statistical analysis*

In the seizure tests, ED_50s _of the essential oil as the mean value with a 95% confidence interval was calculated using the Probit-Regression method (SPSS software, Chicago, IL; version 17.0). In the acetic acid-induced writhing test, ID_50s_ values were calculated using nonlinear regression analysis. In the rest of the experiments, the results were compared using One-way Analysis of Variance (ANOVA), and the differences among the mean values were tested with the Tukey post-test in Graph Pad Prism software (San Diego, CA; version 5.0), when appropriate. The data was presented as the mean ± standard error of the mean (SEM). In all tests, *p *< 0.05 ‎was considered a statistically significant difference. 

## Results

*Memory-enhancing properties of DAG-EO*

**Figure 1:**
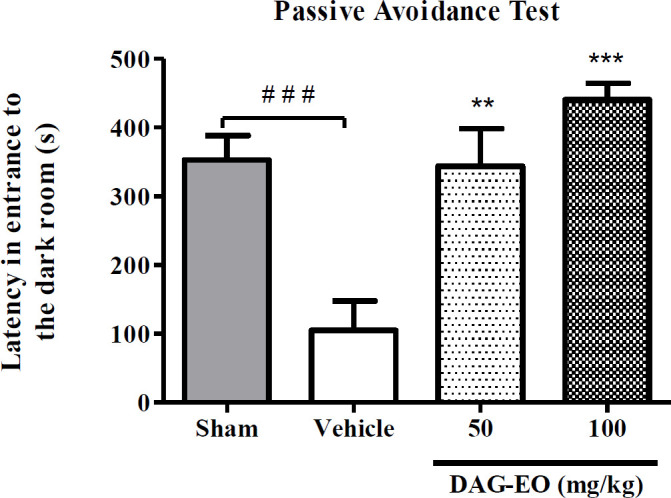
Effect of DAG-EO on avoidance latency in D-galactose-induced memory impairment. All values are expressed as mean ± SEM. ^‎‎^^***^indicate *p*-value < 0.001, ^**^indicates *p*-value < 0.01 ‎compared to the vehicle group, ^###^indicates *p*-value < 0.001 in two defined groups‎; (n = 10) in each group

In the passive avoidance task, a significant reduction in the latency time was observed in the D-galactose-treated group (104.6 ± 33.29 s) compared to the sham group (353.1 ± 35.41 s). This reduction in latency time illustrates that administration of D-galactose at the dose of 100 mg/kg once daily for 49 consecutive days induces memory impairment in mice. This experiment also revealed that the latency in entering the dark room in the groups that received 50 and 100 mg/kg DAG-EO for 7 consecutive days within the last 7 days of animal treatment with D-galactose were significantly lengthened (343.8 ± 44.64 s and 440.8 ± 24.01 s, respectively) as compared with D-galactose-treated mice (Figure 1). 

**Figure 2:**
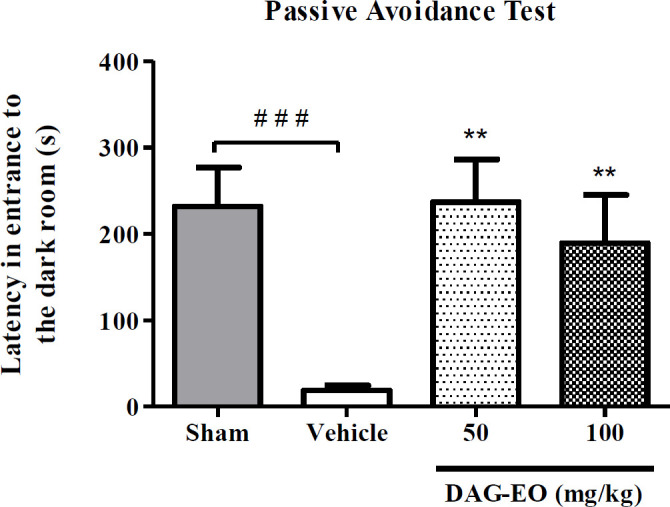
Effect of DAG-EO on avoidance latency in scopolamine-induced memory impairment. All values are expressed as mean ± SEM. ^‎^^**^indicates *p*-value < 0.01 ‎compared to the vehicle group, ^###^indicates *p*-value < 0.001 in two defined groups‎; (n = 10) in each group

Considering the result of the passive avoidance task in the scopolamine-induced memory deficit, the administration of scopolamine remarkably reduced the latency time (18.9 ± 5.5 s) in comparison to the sham group (231.8 ± 35.8 s). This reduction in the latency time was reversed by the administration of DAG-EO at the doses of 50 and 100 mg/kg (237.43 ± 39.43 s and 189.5 ± 47.34 s, respectively) (Figure 2).

**Figure 3:**
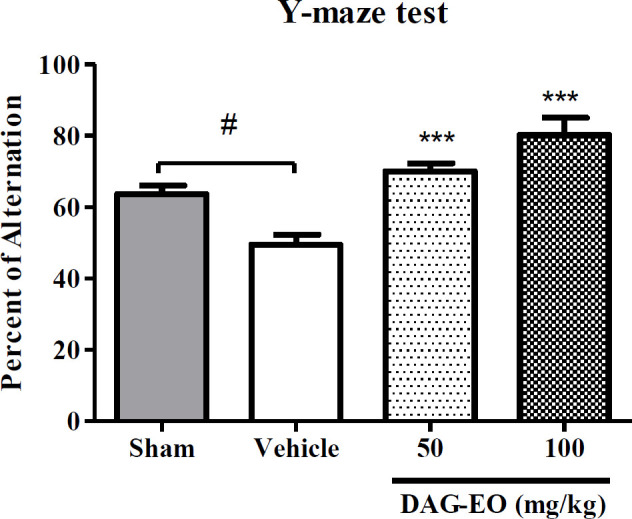
Effect of DAG-EO on the percentage of spontaneous alternation ‎ in D-galactose-induced memory impairment. All values are expressed as mean ± SEM. ^‎^^***^indicates *p*-value < 0.001 ‎compared to the vehicle group, ^#^indicates *p*-value < 0.05 in two defined groups‎; (n = 10) in each group

In the Y-maze test, the percentage of spontaneous alternation was considered as an index of short-term memory to evaluate the effect of DAG-EO on reversing memory impairment induced by D-galactose. As shown in Figure 3, the percentage of spontaneous alternation was reduced in the aging group (49.5 ± 5.1%) comparing to the sham group (64.3 ± 2.4%). The aging induced by D-galactose significantly was improved in the animals which received DAG-EO for 7 days compared with the aging group. The percentage of spontaneous alternations was increased to 70.1 ± 2.3% and 80.3 ± 5.3% in the animals which received DAG-EO 50 and 100 mg/kg, respectively.

*Total activity of animals*

**Figure 4:**
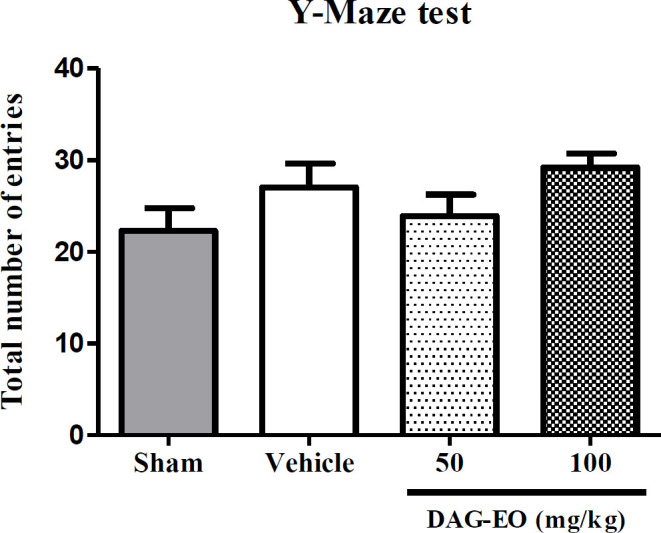
Effect of DAG-EO on the total number of arm entries in the Y-maze test‎. All values are expressed as mean + SEM; (n = 10) in each group

In order to evaluate the effect of different treatments on the total locomotor activity of animals, the number of total arm entries in the Y-maze test was calculated. As shown in Figure 4, no significant difference was found in the total number of entries among the different groups. 

**Figure 5:**
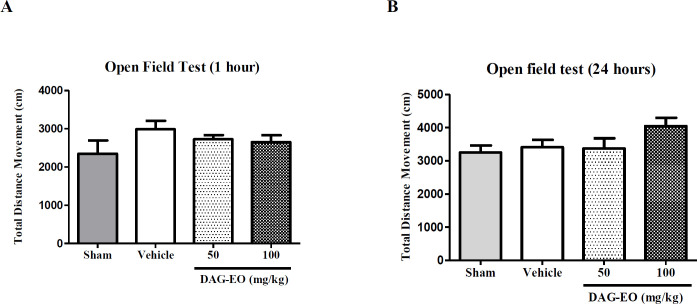
Effect of DAG-EO on the locomotor activity of mice (A) one hour and (B) 24 h after the last administration of DAG-EO‎ in the open field test‎. All values are expressed as mean + SEM. (n = 10) in each group

The locomotor activity of mice was also calculated in the open field test one hour after the last administration of DAG-EO on day 49. The experiment was repeated on day 50 to evaluate the effect of DAG-EO on the locomotor activity of animals after 24 h. Figures 5A and 5B illustrate no significant difference in the total distance movement of DAG-EO treated mice comparing to the vehicle after one hour and 24 h of treatment.

*The hypnotic effect of DAG-EO*

**Figure 6:**
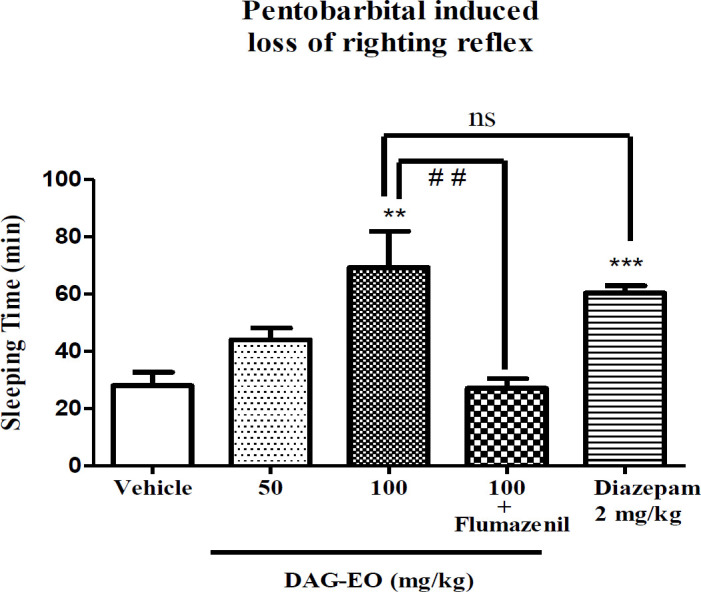
Effect of DAG-EO on the sleeping time in Pentobarbital-induced sleep test. All values are expressed as mean + SEM. ^‎^^***^indicates *p*-value < 0.001 ‎compared to the vehicle group, ^‎^^**^indicates *p*-value < 0.01 ‎compared to the vehicle group, ns indicates no significant and ^##^indicates *p*-value < 0.01 in two indicated groups; (n = 10) in each group

As illustrated in Figure 6, the mice that received DAG-EO (100 mg/kg) had a longer duration of sleep (59.20 ± 12.15 min) comparing to the group that only received vehicle (28.00 ± 4.67 min), but the action of DAG-EO (50 mg/kg) on sleeping time (44.00 ± 13.08 min) was not more notable than vehicle group. Diazepam as the agonist of GABA_A_ receptors increased the duration of sleep in mice to 60.40 ± 2.48 min and showed a significant hypnotic effect as the positive control. There was also no significant difference between the groups that received DAG-EO (100 mg/kg) and diazepam (2 mg/kg). The hypnotic effect of DAG-EO was significantly antagonized by the administration of flumazenil. 

*Anti-seizure effect of DAG-EO*

By administration of DAG-EO and diazepam at different doses, ED_50_ of each agent in protection against the seizure induced by pentylenetetrazole and maximal electroshock was estimated in PTZ and MES models of seizure, respectively (Tables 1 and 2).

*The anti-nociceptive activity of DAG-EO*

Reduction in the number of writhes in the acetic acid-induced writhing test was considered as an anti-nociceptive activity. Comparing the number of writhes in different groups, administration of DAG-EO at the doses of 200 and 400 mg/kg and celecoxib at the doses of 20, 40, and 80 mg/kg significantly decreased the number of writhes compared to the control group. ID_50s_ as the dose of either DAG-EO or celecoxib that reduces the number of writhes to the order of 50% compared to ‎the vehicle group are presented in Table 3.

*Acute toxicity study*

Since some herbs have potent ingredients and should be taken with the same level of caution as pharmaceutical medications, the toxicity of the DAG-EO was estimated in different experiments. Following acute administration of the DAG-EO, the maximum non-fatal dose was 1.5 g/kg, and the median lethal dose (LD_50_) was estimated to be 5.68 (5.26-5.94) g/kg. No significant differences in the animals’ weight gain among the sham (19 ± 6 g), vehicle (21 ± 8 g), 50 mg/kg of DAG-EO (18 ± 6 g) and 100 mg/kg of DAG-EO (21 ± 7 g) groups within 49 days of the experiment were found. 

**Figure 7:**
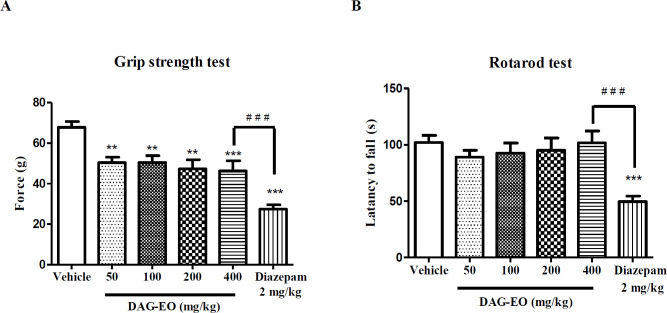
Effect of DAG-EO and diazepam on (A) the grip force and (B) the motor coordination of animals. All values are expressed as mean + SEM. ‎***indicates *p* < 0.001 ‎compared to the vehicle group, ‎**indicates *p* < 0.01 ‎compared to the vehicle group, ‎^# # #^indicates *p* < 0.001 in the two ‎indicated groups; (n = 10) in each group

The acute toxicity of DAG-EO was also evaluated in the grip strength and rotarod tests. In the grip strength test, the mean grip force in animals that received the vehicle was 67.85 ± 2.88 g. DAG-EO at 50 mg/kg (48.38 ± 2.56 g), 100 mg/kg (50.53 ± 3.28 g), 200 mg/kg (47.33 ± 4.53 g), and 400 mg/kg (46.28 ± 5.01 g) significantly affecting neuromuscular strength in mice, but this effect was less than the effect of diazepam (27.44 ± 2.16 g) (Figure 7A). As shown in Figure 7B, no significant difference was observed in the latency to fall in rotarod among the control and treated mice, but the administration of diazepam (2 mg/kg) significantly decreased the latency to fall (49.60 ± 4.84 s) compared to the control group (102.1 ± 6.22 s).

**Table 1:** Effect of DAG-EO, diazepam, and flumazenil on PTZ-induced convulsion in mice

	**PTZ test**	
**Agent**	**Protection (%)**	**ED**_50_ ** (mg/kg)** **(95% Confidence Intervals)**
Vehicle	0	
DAG-EO (100 mg/kg)	10	
DAG-EO (200 mg/kg)	40	
DAG-EO (400 mg/kg)	60	316.22 (229.08-407.38)
DAG-EO (600 mg/kg)	80	
DAG-EO (800 mg/kg)	100	
DAG-EO (800 mg/kg) + Flumazenil	50	
DAG-EO (800 mg/kg) + Naloxone	20	
Diazepam		0.71 (0.52-0.85)

**Table 2:** Effect of DAG-EO, diazepam, and flumazenil on MES-induced convulsion in mice

	**MES test**	
**Agent**	**Protection (%)**	**ED**_50_ ** (mg/kg)** **(95% Confidence Intervals)**
Vehicle	0	
DAG-EO (100 mg/kg)	30	
DAG-EO (200 mg/kg)	60	
DAG-EO (400 mg/kg)	80	169.82 (114.81-239.88)
DAG-EO (600 mg/kg)	100	
DAG-EO (600 mg/kg) + Flumazenil	60	
DAG-EO (600 mg/kg) + Naloxone	30	
Diazepam		1.07 (0.80-1.33)

**Table 3:** Estimated ID_50s _of DAG-EO and celecoxib in the acetic acid-induced writhing test

**Estimated Potency** **ID**_50_ ** (mg/kg)** **(95% Confidence Intervals)**	**Significancy**	**Response (n)** **Mean ± SEM**	**Dose (mg/kg)**	**Agent**
175.1 (152.9 to 200.5)		30.10 ± 2.88	0	DAG-EO
	ns	30.40 ± 2.32	100	
	***	19.10 ± 1.97	200	
	***	14.00 ± 2.16	400	
19.87 (19.09 to 20.64)		30.10 ± 2.88	0	Celecoxib
	ns	30.75 ± 9.16	10	
	*	21.00 ± 5.30	20	
	***	11.50 ± 4.90	40	
	***	4.375 ± 1.32	80	

^***^indicates *p* < 0.001, ‎^*^indicates *p* < 0.05, and ns indicates no significant difference compared to the vehicle group.

## Discussion

Over the past decades, interest in the investigation of new complementary and alternative medicines from natural sources for medicinal and therapeutic purposes has been increased due to various side effects associated with the use of chemical medicines (24). It has been reported that numerous phytochemicals such as alkaloids, glycosides, polyphenols, saponins, and terpenes which present in medicinal plants have various pharmacological effects (25, 26). In the next few decades, herbal medicines will be considered as a good replacement of chemical medicines in the medical system for the management of human diseases due to their potential therapeutic effect, fewer side effects, and economic viability. Therefore, there is a need to discover and characterize potential pharmacological effects of new natural drugs, the exact mechanism(s) of action, and their possible side effects. Since there was some evidence on the therapeutic effects of DAG on some diseases related to the central nervous system (27), the neuro-behavioral properties of DAG-EO were evaluated in different animal models. Furthermore, the possible toxicity and side effects of this essential oil were explored. 

Passive avoidance and Y-maze tests were used to evaluate the memory-enhancing properties of DAG-EO in D-galactose and scopolamine-induced aged mice. Our finding indicated that the administration of the essential oil at different doses could attenuate and treat the memory deficit and learning impairment induced by chronic and acute administration of D-galactose and scopolamine, respectively. Based on the literature review, this is the first study that reveals the prophylaxis and therapeutic effect of sub-acute administration of DAG-EO on D-galactose and scopolamine-induced cognitive deficit. The acetylcholinesterase inhibitory activity of DAG constituents was previously reported by Adhami *et al.* They proposed that due to this effect, improvement in the cognitive function following administration of DAG seems plausible (8). Moreover, it is well established that GABA_A_ receptors modulate acetylcholine release in the basal forebrain (28). Since the administration of DAG-EO had no significant effect on the locomotor activity of mice either in open field test or Y-maze test, it could be concluded that the observed latency in the entrance to the dark room is due to memory-enhancing properties of the essential oil and not due to the reduced locomotor activity of the animals.

The sedative-hypnotic activity of DAG-EO was determined in the pentobarbital-induced sleeping test. This is a classical method for the determination of sedative and hypnotic properties of compounds in behavioral pharmacology. In our study, the acute administration of DAG-EO caused a dose-dependent increase in the duration of hypnosis induced by pentobarbital. As expected, a similar effect was observed following the administration of diazepam. The sedative effect produced by DAG-EO was fully antagonized following the administration of flumazenil as an antagonist of GABA_A_ receptors. Therefore, participation of the GABAergic system in the observed effect could be considered as a possible mechanism of action. As our best of knowledge, this is the first report on the hypnotic activity of DAG-EO and the involvement of the GABAergic system in the observed effect.

In the current study, PTZ as a known chemo-convulsant model and MES as an electro-convulsant induced seizure were used for studying epilepsy which causes the seizure similar to petit mal and grand mal epilepsy, respectively (29, 30). The involvement of the GABAergic complex system and opioid receptors in the anticonvulsant effect was also investigated. Our preliminary study on the anticonvulsive activity of DAG-EO and the possible mechanism of the action revealed promising evidence on the prevention of seizure-induced either by pentylenetetrazole or maximal electroshock. This finding is in agreement with the results of the previous *in-vivo* study conducted by Abizadeh *et al.* and Motevalian *et al.* on DAG extract but in contrast to what Ghasemi *et al.* observed in their *in-vitro* study at the cellular level (31). They reported that exposure of cells to gum alone decreases the neural excitability while it does not produce a significant antiepileptic effect against PTZ-induced epileptiform (7, 32). 

There are several reports on the involvement of GABA_A _and opioid receptors in epilepsy (33-35). It is well established that anticonvulsant agents such as diazepam and phenobarbital directly inhibit the GABA_A_ receptors (36). In addition, it has been shown that the administration of low doses of morphine as a μ-opioid receptor agonist decreases seizure susceptibility (37, 38). There is also a relation between the GABAergic system and the central opioidergic system in the modulation of the seizure (38). As shown in Tables 1 and 2, co-administration of DAG-EO and flumazenil as an antagonist of GABA_A_ receptors antagonized the observed effect in the both PTZ and MES tests. A similar result was obtained by ‎the co-administration of DAG-EO and naloxone as an antagonist of opioid receptors. Since the anticonvulsive activity was highly diminished by opioid and benzodiazepine receptor antagonists, probably both receptors are involved in the observed effect but opioid receptors seem to be more important since the anticonvulsant activity of DAG-EO was antagonized by naloxone much more than flumazenil. 

Writhing test, as a chemical-induced pain model (39), was used for the investigation of the peripheral anti-nociceptive ‎activity of DAG-EO. Analgesic activity of DAG-EO was uncovered in the acetic acid-induced writhing test. The observed activity was in a dose-dependent manner. The highest analgesic activity of DAG-EO was found at the dose of 400 mg/kg, which was comparable to celecoxib. Considering this fact that inflammatory cytokines and autacoids such as interleukins and bradykinins are responsible for peripheral pain, inhibition of pro-inflammatory cytokines might be one of the possible mechanisms of action in the observed effect. Furthermore, the antioxidant activity of DAG-EO is considered as another possible mechanism (9). However, further studies need to be conducted to confirm these hypotheses. 

Although in all experiments, the estimated ED_50__s_ and ID_50s _were below the maximum non-fatal dose, still it is very important to evaluate possible side effects of DAG-EO in different ways. In this regard, the effect of DAG-EO at different doses on the locomotor activity, muscle strength, and motor coordination of animals was evaluated in the open field, grip strength, and rotarod tests, respectively. These tests were chosen because the change in locomotor activity, reduction of muscle strength and impairment of motor coordination have been reported as common side effects of benzodiazepine receptors agonists (40). As illustrated in Figures 5 and 7B, no significant differences were observed in the total distance movement and latency to fall between the control and treated mice. Hence, it could be concluded that obtained results from the passive avoidance test are not affected by the immobility or motor coordination impairment induced by DAG-EO. Although DAG-EO at all tested doses affected the grip strength force and showed muscle relaxant activity, this effect was less than that of diazepam.

## Conclusion

Most of the previous studies have focused on the pharmacological properties of DAG extract, but this is the first study focusing on the biological activity and possible toxicity of the essential oil of DAG. The results of the present study supported that DAG-EO improves memory impairment induced by sub-chronic administration of D-galactose or acute administration of scopolamine through modulation of acetylcholinesterase activity or indirectly by activation of GABA_A_ receptors. Our findings also suggested that DAG-EO possesses a dose-dependent CNS depressant activity such as anticonvulsant, sedative-hypnotic, and anti-nociceptive activities possibly mediated via directly and/or indirectly modulation of GABA_A_ and opioid receptors. The output of the present discussion justifies the traditional use of DAG-EO in the treatment of epilepsy, amnesia, and as a sedative-hypnotic agent but further studies need to be carried out to find out the exact mechanism involved in the observed effects.
